# Ethanol Extract of *Dianthus chinensis* L. Induces Apoptosis in Human Hepatocellular Carcinoma HepG2 Cells *In Vitro*

**DOI:** 10.1155/2012/573527

**Published:** 2012-05-08

**Authors:** Kyoung Jin Nho, Jin Mi Chun, Ho Kyoung Kim

**Affiliations:** Center of Herbal Resources Research, Korea Institute of Oriental Medicine, Daejeon 305-811, Republic of Korea

## Abstract

*Dianthus chinensis* L. is used to treat various diseases including cancer; however, the molecular mechanism by which the ethanol extract of *Dianthus chinensis* L. (EDCL) induces apoptosis is unknown. In this study, the apoptotic effects of EDCL were investigated in human HepG2 hepatocellular carcinoma cells. Treatment with EDCL significantly inhibited cell growth in a concentration- and time-dependent manner by inducing apoptosis. This induction was associated with chromatin condensation, activation of caspases, and cleavage of poly (ADP-ribose) polymerase protein. However, apoptosis induced by EDCL was attenuated by caspase inhibitor, indicating an important role for caspases in EDCL responses. Furthermore, EDCL did not alter the expression of bax in HepG2 cells but did selectively downregulate the expression of bcl-2 and bcl-xl, resulting in an increase in the ratio of bax:bcl-2 and bax:bcl-xl. These results support a mechanism whereby EDCL induces apoptosis through the mitochondrial pathway and caspase activation in HepG2 cells.

## 1. Introduction

Hepatocellular carcinoma (HCC) is the fifth most commonly diagnosed cancer, with more than 1 million deaths reported annually worldwide [1]. Exposure to aflatoxin B1 and infection with hepatitis B virus and hepatitis C virus are high-risk factors for HCC [2–4]. The high prevalence and high death rate require novel strategies for the prevention and treatment of hepatic cancer. Natural products with antitumor activity are a promising approach to cancer prevention.

Plants are valuable sources of bioactive compounds and are used for medicinal purposes in Asia including Korea. Recently, oriental medicine has been the focus of scientific discovery efforts into novel drugs including anticancer agents [5–9]. Several herb-based components and extracts have been reported to reduce tumor growth and inhibit metastasis in the human HCC HepG2 model *in vitro* and *in vivo* [10, 11].

*Dianthus chinensis *L. (Caryophyllaceae, Rainbow pink) is commonly known as “Pae-raeng-ee-kot” in Korea. In Korea, this herb is used as a folk remedy for the treatment of menostasis, gonorrhea, cough, diuretic, and emmenagogue [12]. The chemical components of *Dianthus chinensis *L. are eugenol, phenylethylalcohol [13], melosides A and L [14] and dianchinenosides A, B [15], C, and D [16]. Hypotensive, anthelmintic, intestinal peristaltic, antitumor, and antioxidant activity was documented [12, 13, 17, 18]. However, apoptosis induction by this herb was never reported. The ethnomedical information described above formed the basis for the present study, which was conducted to evaluate the cytotoxic activity and mechanism of action of the ethanol extract of *Dianthus chinensis *L. in HepG2 HCC cells.

## 2. Materials and Methods

### 2.1. Plant and Preparation of Extracts

*Dianthus chinensis *L. was purchased as a dried herb from OmniHerb Co. (Yeongcheon, Korea) and authenticated based on microscopic and macroscopic characteristics by the Classification and Identification Committee of the Korea Institute of Oriental Medicine (KIOM). The dried herb (30.26 g) was extracted twice with 70% ethanol (with 2 h reflux) and the extract was then concentrated under reduced pressure. The decoction was filtered, lyophilized, and stored at 4°C. The yield of dried extract from starting crude material was approximately 18.57% (w/w). The lyophilized powder was dissolved in 10% dimethyl sulfoxide and then filtered through a 0.22 *μ*m syringe filter to create a stock solution. EDCL denotes* Dianthus chinensis *L. ethanol extract. EDCL was diluted in culture medium to the final concentration indicated for each experiment. 

### 2.2. Cell Culture

HepG2 human hepatocarcinoma cells were obtained from the American Type Culture Collection (Manassa, VA, USA). Cells were routinely maintained in Minimum Essential Medium with Earle's Balanced Salts and L-glutamine (MEM/EBSS, HyClone, Logan, UT, USA) supplemented with 10% fetal bovine serum (Gibco BRL, Gaithersburg, MD, USA), 100 U/mL penicillin (Gibco BRL), and 100 *μ*g/mL streptomycin (Gibco BRL) at 37°C in a humidified atmosphere of 5% CO_2_. The culture medium was replaced every 2 days.

### 2.3. Cell Viability Assay

Cells were seeded in 96-well culture plates at a density of 2 × 10^4^ cells/well and allowed to adhere at 37°C for 12 h. The following day, several concentrations of EDCL were added and the cells were further incubated for 48 h. Then, cell viability was measured using the CCK-8 assay. 10 *μ*L CCK-8 reagent was added to each well and incubated for 1 h at 37°C. Cell viability determination was based on the bioconversion of tetrazolium into formazan by intracellular dehydrogenase. Absorbance was measured at 450 nm using a Benchmark Plus Microplate Spectrophotometer (Bio-Rad, Hercules, CA, USA). Cytotoxicity was expressed as a percentage of the absorbance measured in control untreated cells.

### 2.4. Nuclear Staining with Hoechst 33342

Hoechst 33342 (Invitrogen, Eugene, Oregon, USA) staining was used to observe the apoptotic morphology of cells. Briefly, 5 × 10^5^ cells/mL were seeded in six-well plates and incubated for 24 h. Then, the cells were exposed to different concentrations of EDCL (50–400 *μ*g/mL) for 48 h. Next, the cells were collected and fixed with 3.7% formaldehyde in phosphate buffered saline (PBS) for 15 min and stained with Hoechst 33342 (10 *μ*g/mL) at room temperature for 10 min. Finally, after the cells were washed with PBS, morphological changes, including a reduction in volume and nuclear chromatin condensation, were observed by fluorescence microscopy (Olympus Optical, Tokyo, Japan) and photographed at a 400x magnification.

### 2.5. Flow Cytometric Analysis for Measurement of Sub-G1 Phase

Cells were seeded in six-well plates at 1 × 10^6^ cells/well and allowed to attach overnight. After exposure to EDCL, cells were collected, washed twice with ice-cold PBS buffer (pH 7.4), fixed with 80% ethanol at 4°C for 2 h, and then stained with PI/RNase Staining Buffer (BD PharMingen, San Diego, CA, USA) for 20 min in the dark at room temperature. Apoptotic cell analysis was conducted on a FACS Calibur flow cytometer (BD Biosciences, San Jose, CA, USA) and the data were analyzed using the CellQuest software.

### 2.6. Assay of Caspase-3/7, -8, and -9 Activity

Caspase activity was assayed using Caspase-Glo assay kits (Promega, Madison, WI, USA) according to manufacturer protocols. Briefly, cells were seeded at a density of 2 × 10^4^ per well in triplicate wells onto 96-well plates and incubated for 24 h. Afterwards, the cells were exposed to several concentrations of EDCL (50–400 *μ*g/mL) for 48 h or incubated with 180 *μ*g/mL of EDCL for 6–48 h. After exposure to EDCL, culture supernatant (100 *μ*L) was transferred into a white-walled 96-well plate. An equal volume of caspase substrate was added and samples were incubated at room temperature for 1 h. Culture medium was used as a blank control sample and luminescence was measured using an EnVision 2103 Multilabel Reader (PerkinElmer, Wellesley, MA, USA). 

### 2.7. Protein Preparation and Western Blot Analysis

Cells were seeded in six-well plates at 1 × 10^6^ cells/well and allowed to attach overnight. Afterwards, cells were exposed to different concentrations of EDCL for 48 h. Then, the cells were washed with ice-cold PBS twice and lysed with 1X RIPA lysis buffer (50 mM Tris-HCl, pH 8.0, 150 mM NaCl, 1% NP-40, 0.5% sodium deoxycholate, 0.1% SDS and 1 mM Protease Inhibitor Cocktail) for 30 min on ice. Lysates were cleared by centrifugation and supernatants were collected. The total protein content was quantified using the Bradford method. Proteins (30 *μ*g) were mixed with 2X sample buffer, incubated at 95°C for 5 min, and loaded onto 12% polyacrylamide gels. Electrophoresis was performed using the Mini Protean 3 Cell (Bio-Rad). Proteins separated on the gels were transferred onto nitrocellulose membranes (Schueicher & Schell BioScience, Dassel, Germany). Membranes were blocked for 2 h using blocking buffer (10 mM Tris-HCl, pH 7.5, 150 mM NaCl, 0.1% Tween 20, 3% nonfat dry milk) and incubated at 4°C overnight with primary antibody (all antibodies were purchased from Cell Signaling Technology, Beverly, MA, USA). After washing with blocking buffer three times for 30 min, membranes were probed with horseradish peroxidase-conjugated goat anti-mouse immunoglobulin G (IgG) and anti-rabbit IgG (Cell Signaling Technology) for 2 h. The membranes were washed for 1 h (during which the wash buffer was changed three times) with Tris-buffered saline Tween 20 solution and developed with ECL Advance Western Blotting Detection Kit (GE Healthcare, Little Chalfont, Buckinghamshire, UK) using a LAS-3000 luminescent image analyzer (Fuji Photo Film Co. Ltd., Kanagawa, Japan). Western blot signals were quantified and normalized to *β*-actin by densitometry analysis using the Multi-Gauge program of the LAS-3000 imaging system. 

### 2.8. Statistical Analysis

Mean data values are presented with their deviation (mean ± SD) from three independent measurements. Statistical analyses were performed according to Prism 5 program (GraphPad, San Diego, USA). Analysis of variance (ANOVA) was followed by Dunett's test. A value of *P* < 0.05 was considered to be statistically significant.

## 3. Results

### 3.1. Effect of EDCL on HepG2 Cell Growth

**Figure 1:**
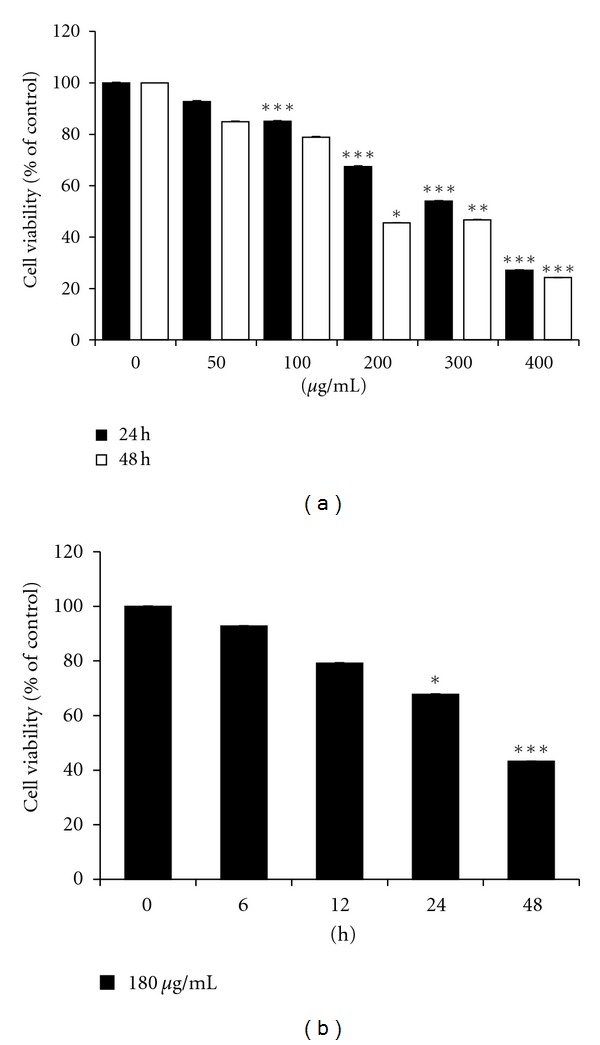
Exposure to EDCL induces growth inhibition in HepG2 cells. (a) Concentration response. Cells were incubated in the presence or absence of several concentrations of EDCL for 24 and 48 h. (b) Time course. Cells were exposed to 180 *μ*g/mL EDCL over time (6–48 h). Cell viability was assessed by CCK-8 assay. The data are expressed as the means ± SD of triplicate samples. *P < 0.05, **P < 0.01, and ***P < 0.001 versus untreated EDCL.

The effect of EDCL on HepG2 cell growth was assessed using the CCK-8 assay.  Figure 1 shows inhibition of HepG2 cell viability by several concentrations (50–400 *μ*g/mL) of EDCL and over time (6–48 h). The results show concentration- and time-dependent inhibition, with IC_50_ values ranging from 314.98 *μ*g/mL (24 h) to 186.64 *μ*g/mL (48 h) (Figure 1).

### 3.2. Effect of EDCL on HepG2 Cell Apoptosis

**Figure 2:**
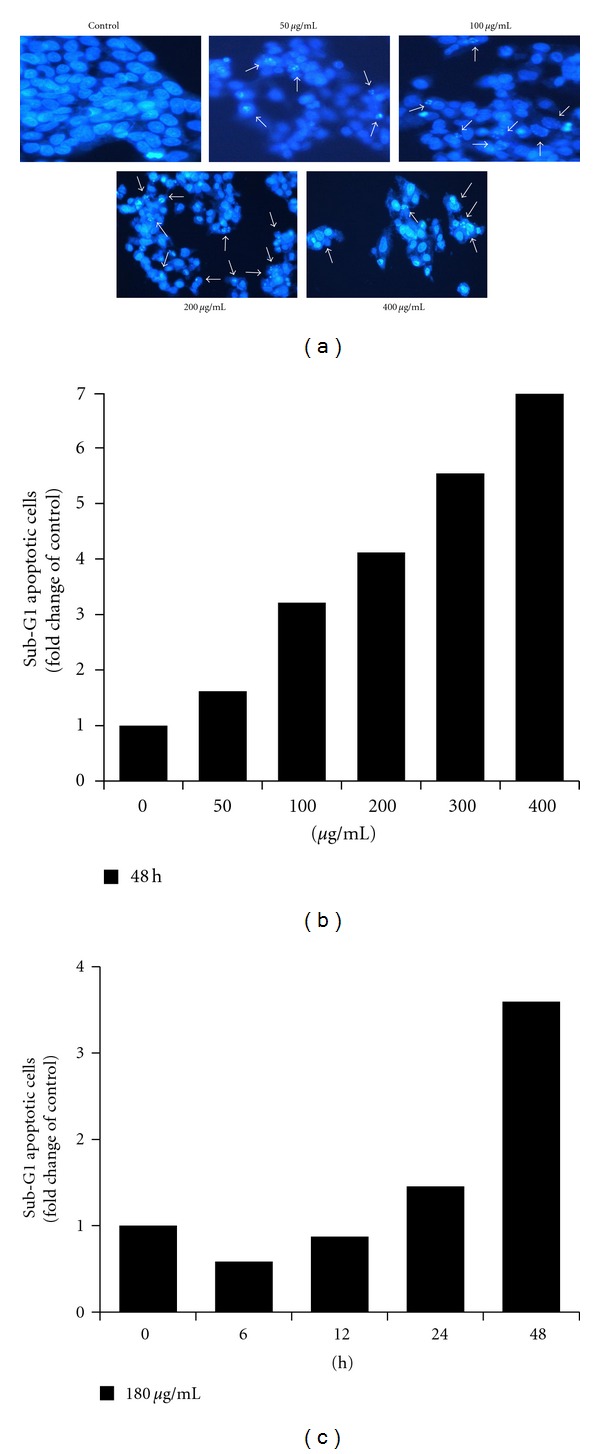
Exposure to EDCL induces apoptosis in HepG2 cells.** (**a) Cells were incubated in the presence or absence of several concentrations of EDCL for 48 h. Hoechst stain showed EDCL-induced chromatin condensation (arrow). Magnification, ×400. (b) Cells were exposed to several concentrations of EDCL for 48 h or (c) exposed to EDCL (180 *μ*g/mL) over time. Apoptosis was measured using PI staining and flow cytometry.

To investigate the effect of EDCL on the morphology of apoptotic cells, Hoechst 33342 staining was conducted. Very few apoptotic cells were observed in the control culture, while the percentage of apoptotic cells in the presence of EDCL increased in an EDCL concentration-dependent manner (Figure 2(a)). The amount of sub-G1 DNA was analyzed to quantify the number of dead cells, since dead cells have a lower DNA content than cells in the G1 phase. Flow cytometric analysis indicated that exposure to EDCL markedly increased the number of sub-G1 phase cells in a concentration- and time-dependent manner (Figures 2(b) and 2(c)).

### 3.3. Effect of EDCL on the Apoptotic Mitochondrial Pathway

**Figure 3:**
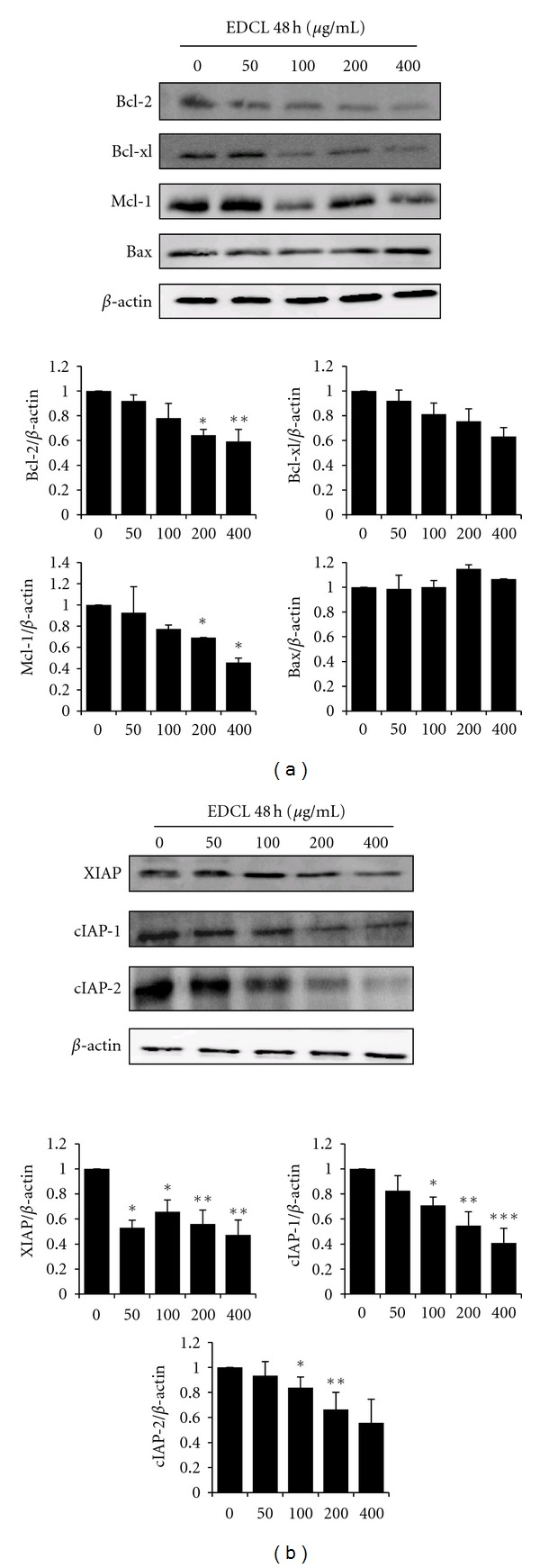
Exposure to EDCL downregulates the expression of Bcl-2 and IAP family members in HepG2 cells. Cells were exposed to several concentrations of EDCL for 48 h. Protein levels were monitored by Western blot analysis. Western blot signals were quantified and normalized to *β*-actin. Values are expressed as means ± SD. *P < 0.05, **P < 0.01, and ***P < 0.001 versus untreated EDCL.

The expression level of Bcl-2 family members interacting directly with mitochondria was studied. Western blotting (Figure 3(a)) revealed that the translational levels of bax expression, a proapoptotic protein, remained virtually unchanged in response to EDCL, whereas bcl-2, bcl-xl, and mcl-1, which are antiapoptotic proteins, were inhibited by exposure to EDCL. These data show that EDCL alters the bax:bcl-2 and bax:bcl-xl ratios in HepG2 cells in a concentration-dependent manner. Since proteins from the IAP family bind to caspases, leading to caspase inactivation in eukaryotic cells, the involvement of the IAP family in EDCL-induced apoptosis was further examined. The results indicated that the levels of IAP family members, such as cellular inhibitor-of-apoptosis protein (cIAP)-1, cIAP-2, and X-linked inhibitor of apoptosis protein (XIAP), were downregulated in HepG2 cells exposed to EDCL in a concentration-dependent manner (Figure 3(b)).

### 3.4. Effect of EDCL on Caspase Activity

**Figure 4:**
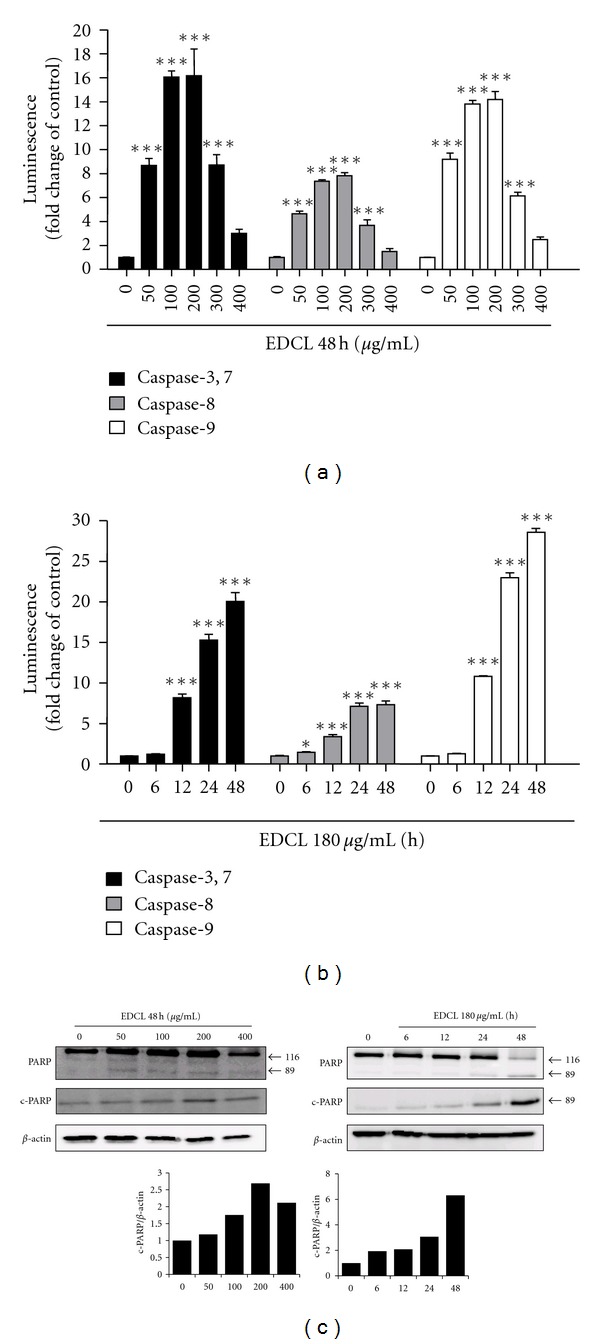
Exposure to EDCL shows activation of caspases and degradation of PARP protein in HepG2 cells. (a) Concentration response. Cells were incubated in the presence or absence of several concentrations of EDCL for 48 h. (b) Time course. Cells were incubated in the presence or absence of 180 *μ*g/mL EDCL for different lengths of time. Upon completion of each exposure time, caspase activity was assessed using a Caspase-Glo assay kits assay, as described in Section 2. The data are expressed as the means ± SD of triplicate samples. *P < 0.05 and ***P < 0.001 versus untreated EDCL. (c) Cells were subjected to Western blot analysis using anti-PARP and anti-c-PARP antibodies. Western blot signals were quantified and normalized to *β*-actin.

To investigate the apoptotic cascade induced by EDCL, HepG2 cells were exposed to several concentrations of EDCL (50–400 *μ*g/mL) for 48 h or incubated with 180 *μ*g/mL of EDCL for 6–48 h, after which caspase-3/7, -8, and -9 activity was measured. The level of caspase activation in HepG2 cells exposed to EDCL was compared to that of control untreated cells arbitrarily set to 1.0. Results showed that EDCL markedly increased caspase-3/7, -8, and -9 activity, with maximum increase activity at 200 *μ*g/mL. Results also showed that caspase activity increased over time in response to 180 *μ*g/mL EDCL (Figures 4(a) and 4(b)). At the concentration of 200 *μ*g/mL, the activity of caspase-3/7, caspase-8, and caspase-9 increased by 16.16-, 7.80-, and 14.17-fold, respectively. Furthermore, EDCL induced the degradation of poly (ADP-ribose) polymerase (PARP, 116 kDa), which is a protein substrate of caspase-3, and PARP cleavage fragments (89 kDa) increased over time (Figure 4(c)).

### 3.5. Effect of Caspase Inhibitor on EDCL-Induced Apoptosis in HepG2 Cells

**Figure 5:**
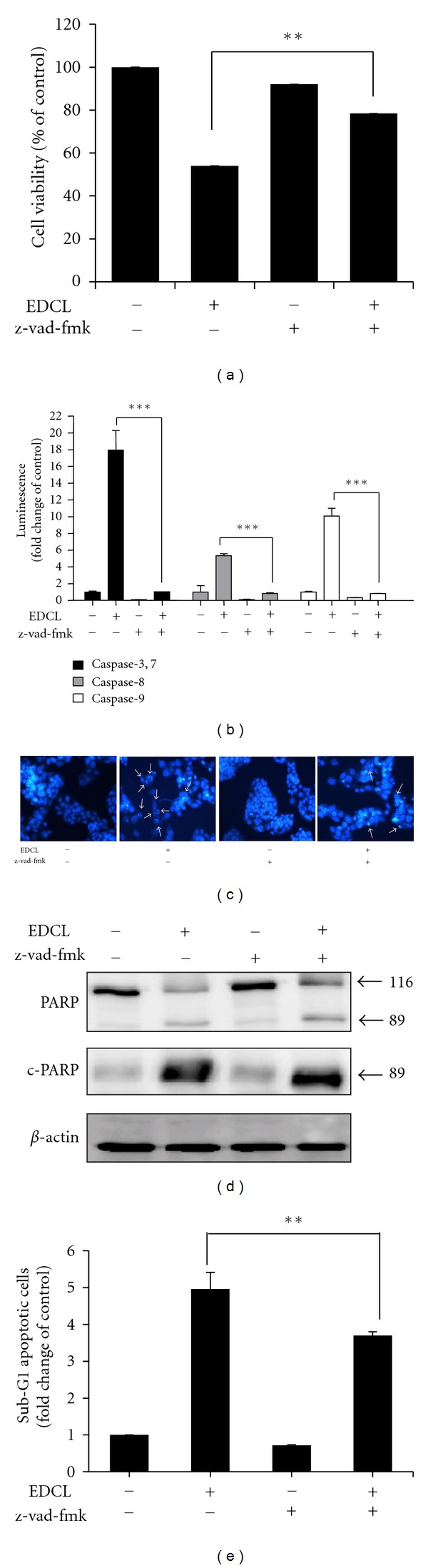
Caspase inhibition prevents EDCL-induced apoptosis in HepG2 cells. Cells were incubated in the presence or absence of z-vad-fmk for 1 h before being exposed to EDCL (180 *μ*g/mL). (a) After 48 h of incubation with EDCL, cell viability was assessed using the CCK-8 assay and (b) caspase activity was measured. (c) Hoechst staining shows EDCL-induced chromatin condensation (arrow). Magnification, ×400. (d) Cells were subjected to Western blot analysis using anti-PARP and anti-c-PARP antibodies. (e) Cells were evaluated for sub-G1 DNA content by flow cytometry. The data are expressed as the means ± SD of triplicate samples. *P < 0.05, **P < 0.01, and ***P < 0.001 versus EDCL+z-vad-fmk.

To confirm that caspase activation is a key step in EDCL-induced apoptosis, HepG2 cells were pretreated with z-vad-fmk (80 *μ*M), a broad-spectrum caspase inhibitor, for 1 h, and then subsequently exposed to 180 *μ*g/mL EDCL for 48 h. As shown in Figure 5(a), z-vad-fmk did not affect cell viability but inhibited the antiproliferative activity of EDCL. EDCL strongly stimulated caspase protease activity and pretreating cells with z-vad-vmk nearly abolished EDCL-induced caspase activity (Figure 5(b)). Furthermore, blockade of caspase activity by z-vad-vmk prevented EDCL-induced chromatin condensation (Figure 5(c)), PARP degradation (Figure 5(d)), and increase in sub-G1 population (Figure 5(e)). These results clearly show that EDCL-induced apoptosis is associated with caspase activation.

## 4. Discussion

During the last decade, a considerable amount of research has focused on cancer cell apoptosis. Apoptosis, or programmed cell death, is the major control mechanism by which cells die if DNA damage is not repaired [19]. Apoptosis is also a critical protective mechanism against carcinogenesis, eliminating damaged cells or cells proliferating abnormally in response to carcinogens [20]. Therefore, induction of apoptotic cell death is a promising emerging strategy for the prevention and treatment of cancer [21]. The results of the present study clearly demonstrate that EDCL suppressed HepG2 cell viability by inducing apoptosis. After exposure to EDCL, chromatin condensation and apoptotic bodies were clearly observed. These results suggest that HepG2 cells exposed to EDCL undergo typical apoptosis. Furthermore, flow cytometric analysis after propidium iodide staining confirmed EDCL-induced apoptosis in HepG2 cells.

Members of the Bcl-2 family of proteins, such as bcl-2, bcl-xl, mcl-1, and bax, are the most prominent actors in controlling the release of cytochrome c and in mitochondria-mediated apoptosis [22]. Thus, it has been suggested that the ratio between the level of proapoptotic bax protein and the level of antiapoptotic bcl-2 protein determines whether a cell responds to an apoptotic signal [23]. In this study, EDCL did not alter the expression of bax in HepG2 cells but did selectively downregulate the expression of bcl-2 and bcl-xl, resulting in an increase in the ratio of bax:bcl-2 and bax:bcl-xl.

The execution of cellular demolition in apoptosis is also carried out by caspases [24]. The caspase family of proteins is one of the main executors of the apoptotic process. Caspases belong to a group of enzymes known as cysteine proteases and exist within the cell as inactive proforms or zymogens. These zymogens can be cleaved to form active enzymes following the induction of apoptosis. The IAP family of proteins blocks apoptosis by directly inhibiting at least two members of the caspase family of cell death proteases, caspase-3, and caspase-7. XIAP, cIAP-1, and cIAP-2 can prevent the proteolytic processing of procaspase-3, -6, and -7 by blocking the cytochrome c-induced activation of procaspase-9 [24, 25]. Studies have shown that exposure of HepG2 cells to EDCL caused proteolytic activation of caspases and down-regulation of XIAP, cIAP-1 and cIAP-2. The enzyme poly(ADP-ribose) polymerase, or PARP, was one of the first proteins identified as a substrate for caspases. PARP is involved in repair of DNA damage and functions by catalyzing the synthesis of poly (ADP-ribose) and by binding to DNA strand breaks and modifying nuclear proteins. PARP helps cells maintain viability, and the cleavage of PARP facilitates cellular disassembly and serves as a marker for cells undergoing apoptosis [26, 27]. In the present study, we examined whether the PARP protein, a substrate of caspase-3 [28], was cleaved in cells exposed to EDCL. As expected, PARP was clearly degraded in a concentration- and time-dependent manner that correlated with caspase activation. Under the same experimental conditions, z-vad-fmk prevented EDCL-induced apoptosis by blocking caspase activation. These data indicate that caspases are the key molecules mediating EDCL-induced apoptosis in HepG2 cells.

In conclusion, this study clearly demonstrates that EDCL strongly inhibits cell proliferation and induces apoptosis in HepG2 cells. EDCL induced apoptosis through the mitochondrial pathway, involving the activation of caspase-3/7, -8, and -9, the down-regulation of antiapoptotic proteins, and the degradation of PARP protein. Because induction of apoptosis is thought to be a suitable anticancer therapeutic mechanism, these results confirm the potential of EDCL as a chemotherapeutic agent in human hepatocellular carcinoma cells. *In vivo* studies are needed to fully establish the potential of EDCL as a chemopreventive and therapeutic agent in cancer.
